# A kind route from grounding to fundamentality

**DOI:** 10.1007/s11229-021-03163-y

**Published:** 2021-05-16

**Authors:** Fabrice Correia

**Affiliations:** Department of Philosophy, University of Geneva, Rue De-Candolle 5, 1211 Geneva 4, Switzerland

**Keywords:** Fundamentality, Relative fundamentality, Absolute fundamentality, Grounding

## Abstract

I offer an account of fundamentality for facts in terms of metaphysical grounding. The account does justice to the idea that whether a fact is absolutely fundamental, and whether a fact is more fundamental than, or as fundamental as, another fact, are a matter of where in a grounding-induced hierarchy of *kinds* of facts these facts appear.

## Introduction

The notion of (fact) fundamentality comes in two varieties, absolute and relative. Absolute fundamentality is at work when we say that a fact is fundamental *tout court*. Relative fundamentality itself comes in two varieties: there is the notion of *being more fundamental than* and that of *being as fundamental as*. It is natural to think—and it actually has been thought—that (metaphysical) grounding can be used to characterise a notion of fundamentality of each variety.^1^ Thus, many philosophers have recently held the view that one way for a fact to be absolutely fundamental is for it to be ungrounded.^2^ Two of these philosophers, Karen Bennett (2017) and Jonas Werner (forthcoming), have proposed characterisations of relative fundamentality in terms of grounding, and I did the same in Correia forthcoming.^3^

Werner rightly points out that Bennett’s characterisation does not capture a unified notion. At least part of the reason is that it merges two quite different ideas: (i) the idea that the fundamentality status of a fact—that is, whether it is fundamental *tout court*, and whether it is more fundamental than, or as fundamental as, other facts—is a matter of *how far, ground-theoretically speaking, this fact is from the ungrounded facts*, and (ii) the idea that the fundamentality status of a fact is a matter of *where, in a grounding-induced hierarchy of kinds of facts, this fact appears*.^4^ Werner sets himself the task of characterising a notion of fundamentality that does justice to the first idea, and I also do it in Correia forthcoming (I actually characterise *two* such notions there). In the present paper, I set myself the task of characterising a notion of fundamentality that does justice to the second idea.^5^

The plan is as follows. Section 2 deals with preliminary considerations about grounding and relative fundamentality. In Sect. 3, I present Bennett’s account of the relation of *being more fundamental than* (hereafter: <–*fundamentality*), of the relation of *being as fundamental as* (hereafter: = –*fundamentality*) and of absolute fundamentality in terms of grounding, and I give some reasons to reject her account of the first relation. In Sect. 4, I clarify a bit the conception of fundamentality I am interested in. In Sect. 5, I put forward my own account of <–fundamentality and I argue that it is superior to Bennett’s. In Sect. 6, I deal with the sister accounts of = –fundamentality and absolute fundamentality.

## Preliminary considerations on grounding and fundamentality

I will assume that grounding has the following features:

- It is a *relation*, that is, it is expressed by means of a relational predicate, whose *relata* are states of affairs or propositions. I will not make any assumption about which of the two options is correct. It is understood that the *relata* of relative fundamentality and the bearers of absolute fundamentality belong to the same category as the *relata* of grounding, whichever it may be. I will use ‘facts’ as a label for the relevant category of entities.
- It is *factive*, that is, it relates only *obtaining* states of affairs or *true* propositions.
- It is *many-one*, that is, what is grounded is always one fact, and what grounds something can be one or several facts taken together.
- It is *transitive*—to be more accurate, it obeys the following “cut principle”:^6^

For every indexed family of pluralities of facts (Δ_i_)_i∈I_, every indexed family of facts with the same index set (F_i_)_i∈I_, every plurality of facts Γ and every fact G, if the F_i_s together with the members of Γ ground G, then provided that the members of Δ_i_ ground F_i_ for all i ∈ I, the members of the Δ_i_s together with the members of Γ also ground G.

I will also understand ‘partially grounds’ according to the following standard definition:

Fact F partially grounds fact G ≡ G is grounded in F, or in a plurality of facts that comprises F.^7^

Thus, ‘partial’ is used in a loose sense: full grounds count as partial grounds. Given the definition, (d) above entails that partial grounding is transitive.

Assumption (a) is dispensable. The discussion could be run in much the same way on the assumption that grounding is expressed by means of a sentential operator rather than by means of a predicate (and that the same holds of both relative and absolute fundamentality).^8^ The reason for assuming the predicate mode of expression is just convenience: going for an operator regimentation of grounding would force one to appeal to higher-order quantifiers where the predicate regimentation only requires first-order quantifiers over facts, and first-order quantification is more familiar than higher-order quantification.

Assumption (b) is also dispensable: I could have worked with a non-factive notion of grounding throughout. However, given that relative fundamentality and absolute fundamentality are themselves factive, working with a non-factive notion of grounding would have forced me to often slightly complicate the formulation of principles and characterisations by adding conditions to the effect that certain states of affairs obtain / certain propositions are true. Note that since given a non-factive notion of grounding, a corresponding factive notion is easily definable in terms of it, assumption (b) is not ideologically demanding.^9^

By contrast, the discussion to come will substantially rely on assumptions (c) and (d), at some points at least. The alternative to (c) is the very unorthodox view that grounding is many-many: what does the grounding can be one or several facts taken together, and what is grounded can also be one or several facts taken together.^10^ Many-many grounding is far from being well understood, and it is for this reason that I leave it aside. The view that grounding obeys the cut principle is hard to reject and has indeed hardly ever been contested.^11^ Hence, having (d) on board is not a very substantial move—or so I take it. That said, the fact that (c) and (d) are very plausible and that they are part of grounding orthodoxy makes it perfectly appropriate to take them on board. However, the discussion to come can certainly serve as a useful basis for the study of the connections between grounding and fundamentality on the assumption that (c) or (d) should be rejected.

Finally, let me stress that I will take as a constraint on a correct account of <–fundamentality and = –fundamentality that it should predict that the former relation is both transitive and irreflexive (and hence asymmetric) and that the latter relation is an equivalence relation, i.e., reflexive, symmetric and transitive. That <–fundamentality and = –fundamentality have the respective features sounds pre-theoretically obvious indeed.

## Bennett on fundamentality

Bennett (2017) takes grounding to be but one member of a large class of “building relations”, and she proposes a characterisation of <–fundamentality, = – fundamentality and absolute fundamentality indexed to members of this class. Given my aims, I will from now on pretend that she focuses on grounding.

Bennett proposes to characterise the three notions under focus as follows (I use her labels for the first characterisation; throughout the paper, I use ‘F’, ‘G’ and the like as variables for facts and ‘${\mathscr{K}}$’, ‘ℒ’ and the likes for variable for kinds of facts):^12^

(MFT) F *is more fundamental than* G ≡ at least one of the following holds:

1. F is fewer grounding steps away from the ungrounded fact(s) that terminate its unique chain than G is from the ungrounded fact(s) that terminate its unique chain;
2. F partially grounds G;
3. F stands in the ancestral of partial grounding to G;^13^
4. F is ungrounded while G is grounded;
5. F belongs to some kind ${\mathscr{K}}$ and G belongs to some kind ℒ such that

- neither ${\mathscr{K}}$ nor ℒ includes both grounded and ungrounded members, and
- G does not belong to ${\mathscr{K}}$ and F does not belong to ℒ, and
- ℒs are typically or normally grounded in ${\mathscr{K}}$s.

(AFA) F *is as fundamental as* G ≡ for all facts F*, (i) F* is more fundamental than F iff F* is more fundamental than G, and (ii) F is more fundamental than F* iff G is more fundamental than F*.
(F) F *is fundamental* ≡ F is ungrounded.

As I stressed in the introduction, (F) corresponds to a widespread conception of absolute fundamentality. (AFA) is a very natural characterisation of = –fundamentality if we suppose given <–fundamentality. Bennett credits Jon Litland with the idea of this characterisation. Bennett’s substantial contribution here is (MFT).

If I were happy with (MFT), I would not have anything to say against (AFA) or (F), and I would therefore be happy with the whole Bennettian package. However, I find (MFT) unsatisfactory, for many reasons indeed. Let me here mention three. (I have further objections which concern matters of detail, but they can be circumvented by appropriately modifying the account; see Correia forthcoming for objections linked to clause (1).)

I. (MFT) does not seem to capture, or even to be close to capturing, a unified notion. One cannot help but have the feeling that in formulating (MFT), Bennett tried to do justice to a bunch of different pre-theoretic intuitions that sometimes pull in quite different directions, sometimes even in opposite directions. Werner (forthcoming) also highlights the disunity of the account, pointing to the fact that while clauses (1)-(4) are purely ground-theoretic, clause (5) crucially invokes the metaphysical notion of a kind. As it turns out, Bennett is sympathetic to the view that our pre-theoretic conception of <–fundamentality, taken as a whole, may be incoherent (2019: 332; see also 2017: 161). Yet she seems to think that highly disjunctive characterisations in the vicinity of (MFT)—if not (MFT) itself—can capture valuable notions. I doubt it. The right thing to do, or so I believe, is to rather look for accounts of more unified notions, even if this means that they may only very partially fit with our pre-theoretic conception.
II. My second reason for dissatisfaction concerns the presence of the phrase ‘typically or normally’ in clause (5). Bennett does not say enough to fix its meaning. She explains that in this context, normality should probably not be understood in the standard, statistical sense, and she refers to Millikan (1989) for an example of a non-statistical conception (Bennett, 2017: p. 159, fn. 20). Yet I very much doubt that Millikan’s teleological notion of normality, whatever its merits for theorising about biological functions, is the right notion to invoke in the present context. And so, I am left wondering how clause (5) should be understood.
III. The view that (5) is a sufficient condition for a fact F to be more fundamental than a fact G yields unwanted results. Suppose there are the kind *mental fact* and the kind *biological fact*. Suppose that mental facts are grounded in biological facts, and that biological facts are grounded (say, in physical facts). Then thanks to clause (5), (MFT) predicts that (A) every biological fact is more fundamental than every mental fact. No problem yet. But suppose there are the kind *disjunction of conjunctions of atomic facts* and the kind *conjunction of atomic facts* and assume, as orthodoxy about the logic of grounding has it,^14^ that every fact of the first kind is grounded in some fact(s) of the second kind, and that every fact in the second kind is grounded in atomic facts. Then thanks to clause (5), (MFT) predicts that (B) every conjunction of atomic facts is more fundamental than every disjunction of conjunctions of atomic facts. Now we have a problem. For let F be a mental fact that is a conjunction of atomic mental facts, and let G be a biological fact that is a disjunction of conjunctions of atomic biological facts. Then by (A), G is more fundamental than F, and by (B), F is more fundamental than G. But since <–fundamentality is asymmetric, this is impossible.

Some might protest against the previous argument, on the grounds that the kinds *disjunction of conjunctions of atomic facts* and *conjunction of atomic facts* are artificial and that Bennett’s (5) should be understood as quantifying only over non-artificial kinds. I reply in three steps.

First, Bennett says almost nothing about which collections of facts (or objects more generally) she takes to correspond to kinds. She is aware that some restrictions are needed in the context of the formulation of clause (5) in (MFT), but the restrictions she identifies boil down to those expressed by subclauses (a) and (b) in clause (5) (see Bennett, 2017: pp. 159–160). So far as I can see, nothing in what she says rules out the possibility that there be kinds such as *disjunction of conjunctions of atomic facts* and *conjunction of atomic facts*.

Second, it seems to me reasonable to hold that ‘conjunction of atomic *sentences*’ and ‘disjunction of conjunctions of atomic *sentences*’ are phrases that pick out non-artificial collections of sentences. Such phrases are constantly used in logic textbooks, e.g., to pick out collections of sentences that are useful for certain purposes (for defining conjunctive and disjunctive normal forms, for instance). If this is reasonable, then it should be likewise reasonable to hold that the phrases ‘conjunction of atomic *facts*’ and ‘disjunction of conjunctions of atomic *facts*’ pick out non-artificial collections of facts.

Third, let me add a further argument against Bennett’s (5) which follows the same pattern. It invokes, not ground-theoretic connections stemming from logical form, but ground-theoretic connections stemming from the determinable / determinate relation, and it might for this reason be considered more convincing. Let me define the following kinds:

- MACRO = the kind *macrophysical fact*
- SWAN = the kind *fact about swans*
- DETERMINATE = the kind *fact of type [x is F]*, where *being F* is a determinate of *being coloured*
- DETERMINABLE = the kind *fact of type [x is coloured]*

Assume with grounding orthodoxy that every fact in DETERMINABLE is grounded in some fact in DETERMINATE and assume that every fact about swans is grounded in some macrophysical facts. Finally assume that none of these kinds comprise both grounded and ungrounded facts. Then consider the fact [W is white], where W is a regular swan, and the fact [C is coloured], where C is a given surface. Bennett’s clause (5) predicts that [W is white] is more fundamental than [C is coloured] (due to the connection between DETERMINATE and DETERMINABLE) and that the converse holds as well (due to the connection between MACRO and SWAN). Hence, we have again a violation of the asymmetry of <–fundamentality.^15^

## The kind-theoretic conception of fundamentality

My first objection against (MFT) was that it merges different conceptions of <–fundamentality. In the introduction, I stressed that Bennett’s account of fundamentality merges at least two different ideas, the idea that the fundamentality status of a fact is a matter of how far, from a ground-theoretic point of view, the fact is from the ungrounded facts, and the idea that the fundamentality status of a fact is a matter of where, in a grounding-induced hierarchy of kinds of facts, the fact is located. The first idea as applied to <–fundamentality is captured (if only imperfectly) in (MFT) by clause (1), the second idea by clause (5). As I also stressed in the introduction, my aim in this paper is to characterise a notion of fundamentality that meshes well with the second idea. What is, more precisely, the conception of fundamentality I am after—the *kind-theoretic* conception, as I will call it?

This conception involves the following two ideas:

- There is a relation—that I will call *foundation*—between *kinds* of facts which is definable in ground-theoretic terms and which captures the idea that some kinds of facts—the founded facts—metaphysically “arise from” some other kinds of facts—the kinds that found them.
- The fundamentality status of a *fact*—whether it is fundamental, and whether it is more fundamental than, or as fundamental as, other facts—stems from the foundational links between kinds of facts.

These ideas are neatly (although not adequately, see Sects. 3 and 5.1) illustrated by clause (5) in Bennett’s (MFT): subclauses (a) and (c) jointly characterise a certain sort of ground-theoretic connection between two kinds ${\mathscr{K}}$ and ℒ, and clause (5) as a whole says that fact F is more fundamental than fact G when there are kinds ${\mathscr{K}}$ and ℒ connected in the way in question such that F is in ${\mathscr{K}}$ but not in ℒ and G is in ℒ but not in ${\mathscr{K}}$.

A further important idea involved in the kind-theoretic conception of fundamentality is the following:

- A fact may be more fundamental than another fact even though these two facts are ground-theoretically completely disconnected.

To illustrate, consider the fact F_0_ that a given cell undergoes meiosis and the fact G_0_ that a given person experiences an acute pain, where the cell is in a remote galaxy and the person is somewhere on Earth.^16^ It makes perfect sense, on the kind-theoretic conception of fundamentality, to claim that F_0_ is more fundamental than G_0_, even though (we may assume) F_0_ and G_0_ are not ground-theoretically connected. This is also neatly illustrated by clause (5) in (MFT): if we assume that all mental facts are grounded in biological facts, that all biological facts are grounded (say, in physical facts), that F_0_ belongs to the kind *biological fact* but not to the kind *mental fact*, and finally that G_0_ belongs to the latter kind but not to the former kind, then the clause predicts that F_0_ is more fundamental than G_0_.

A few words about the notion of a *kind of facts* that is involved in the kind-theoretic conception of fundamentality are in order before moving on to the next section. The sort of account that I intend to formulate arguably requires to impose some restrictions on which collections of facts correspond to kinds of facts. This can be illustrated with the view that clause (5) in (MFT) is sufficient for <–fundamentality. Suppose there are the kind *mental fact* and the kind *biological or mathematical fact*. Suppose that the mental facts are grounded in biological facts, and that the biological facts and the mathematical facts are grounded (perhaps ultimately in divine facts, or in physical facts and logical facts, respectively). Then if clause (5) in (MFT) is sufficient for <–fundamentality, every mathematical fact is more fundamental than every mental fact. Of course, this is something that one *can* hold. But this is certainly not something that one *must* hold, even if one accepts the assumptions that were made.

This objection to the view that clause (5) is sufficient for <–fundamentality will plausibly be blocked if we require that the kinds of facts appealed to in the clause should be *natural*: it is indeed plausible to holds that the kind *biological or mathematical fact* is non-natural, on any reasonable account of what a natural kind of facts is. Similar objections are likely to be effective against any implementation of the kind-theoretic conception of fundamentality if non-natural kinds of facts are not ruled out. For that reason, I henceforth assume that the kinds of facts that the kind-theoretic conception invokes are natural.

What is it for a kind of facts to be natural in the relevant sense? I have in mind something like Lewis’ (1983) notion of naturalness. A natural kind of facts is a kind that is non-artificial, non-gerrymandered, a kind whose members are genuinely similar. I do not want to be too specific about the characterisation of naturalness, because I take it that the kind-theoretic conception of <–fundamentality should be compatible with various views about the notion, ideally with any coherent view. However, let me emphasise that ‘natural’ in the relevant sense should not be equated with ‘perfectly natural’ à la Lewis (1983) or with ‘structural’ à la Sider (2011). It is understood by these philosophers that what is perfectly natural or structural is fundamental. Yet, as I see things, room should be left for the possibility that highly non-fundamental kinds of facts be natural. It should be possible to hold, for instance, that the kind *mental fact* and the kind *logically complex fact* both count as natural in the relevant sense even though they are not perfectly natural or structural.

In the next section, I propose an account of <–fundamentality that is in line with the kind-theoretic conception of fundamentality, and I do the same in the following section with = –fundamentality and absolute fundamentality.

## A kind-theoretic account of <–fundamentality

Taking clause (5) in Bennett’s (MFT) seriously, the following characterisations of foundation and <–fundamentality suggest themselves:

(Foundation-1) ${\mathscr{K}}$ *founds* ℒ ≡ (A) members of ℒ are typically or normally grounded in some members of ${\mathscr{K}}$, and (B) neither ${\mathscr{K}}$ nor ℒ contains both grounded and ungrounded facts.

(<–Fundamentality-1) F *is more fundamental than* G ≡ for some ${\mathscr{K}}$ and ℒ such that F ∈ ${\mathscr{K}}$, F ∉ ℒ, G ∈ ℒ and G ∉ ${\mathscr{K}}$, ${\mathscr{K}}$ founds ℒ.

Given my second objection to (MFT), one gets a more satisfactory characterisation of foundation if one removes the phrase ‘typically or normally’ in (Foundation-1). If we do so, then condition (B) can be simplified since the condition that ℒ does not contain both grounded and ungrounded facts is automatically satisfied. The resulting characterisation thus goes as follows:

(Foundation-2) ${\mathscr{K}}$ *founds* ℒ ≡ (A) every member of ℒ is grounded in some members of ${\mathscr{K}}$, and (B) ${\mathscr{K}}$ does not contain both grounded and ungrounded facts.

Of course, the resulting characterisation of <–fundamentality is subject to my objection against the view that (MFT)’s clause (5) is sufficient for a fact F to be more fundamental than a fact G, namely objection (III). But let me for a moment put <–fundamentality aside and focus first on the characterisation of foundation.

### Foundation

Foundation, remember, is supposed to capture the idea that some kinds metaphysically arise from other kinds. This “generative” character of the relation makes (Foundation-2) inadequate. One reason is the presence of condition (B). Bennett’s argument for having condition (a) in (MFT)’s clause (5) turns out to be an argument for having the weaker condition that kind ℒ does not contain both grounded and ungrounded facts; she does not argue that kind ${\mathscr{K}}$ should also be required to satisfy this condition (Bennett, 2017, pp. 159–160). Be that as it may, having condition (B) in the characterisation of foundation is objectionable. It should be possible to hold, say, *both* that the kind *microphysical fact* generates, in the intended sense, the kind *thermodynamical fact*, *and* that the kind *microphysical fact* contains both ungrounded members and grounded members (for instance conjunctions of ungrounded microphysical facts).

Another reason to be dissatisfied with (Foundation-2) is that it does not guarantee that foundation is irreflexive. Since foundation is a generative relation, it should arguably have that property. Counterexamples to irreflexivity are actually not hard to find. Suppose that that every member of the kind *fact about physical entities* is grounded in some members of that same kind.^17^ (Foundation-2) then predicts that the kind *physical fact* founds itself. Note that this counterexample also applies to the characterisation obtained from (Foundation-2) by dropping condition (B).

In the light of these objections, it is natural to suggest the following alternative characterisation of foundation:

(Foundation-3) ${\mathscr{K}}$ *founds* ℒ ≡ (A) every member of ℒ is grounded in some members of ${\mathscr{K}}$, and (B) ${\mathscr{K}}$ ≠ ℒ.

But this suggestion will not do either. Granted that every member of the kind *thermodynamical fact* is grounded in some members of the kind *microphysical fact*, (Foundation-3) predicts that the kind *thermodynamical fact* is founded in the kind *physical fact*. This sounds bad given the generative character of foundation: if kind ℒ arises from kind ${\mathscr{K}}$, then surely ℒ cannot be a subkind of ${\mathscr{K}}$.

What about, then, replacing (B) by the condition that and ℒ do not overlap, i.e., that no fact belongs to both ${\mathscr{K}}$ and ℒ ? The resulting characterisation, namely

(Foundation-4) ${\mathscr{K}}$ *founds* ℒ ≡ (A) every member of ℒ is grounded in some members of ${\mathscr{K}}$, and (B) ${\mathscr{K}}$ and ℒ do not overlap,

escapes all the previous objections. Yet it is still problematic, because it does not guarantee that foundation is asymmetric. Given that foundation is a generative relation, it should arguably have that property. Here is a counterexample to asymmetry. Suppose the kind *mental fact* comprises infinitely many subkinds M_1_, M_2_, …, that likewise kind *physical fact* comprises infinitely many subkinds P_1_, P_2_, …, that the kind *mental fact* does not overlap the kind *physical fact*, and finally that for any positive integer n, every member of M_n_ is grounded in some member of P_n_ and every member of P_n_ is grounded in some members of M_n+1_. This assumption is exotic, for sure, but it does not seem incoherent. Given this assumption, (Foundation-4) predicts that the kind *mental fact* both founds and is founded in the kind *physical fact*. Note that since these kinds are distinct, the objection also affects (Foundation-3).

In order to escape this objection, I suggest that we strengthen the defining condition in (Foundation-4) by requiring that no member of ℒ helps ground some member of ${\mathscr{K}}$. The resulting characterisation, namely

(Foundation) ${\mathscr{K}}$ *founds* ℒ ≡ (A) every member of ℒ is grounded in some members of ${\mathscr{K}}$, (B) no member of ${\mathscr{K}}$ is partially grounded in some member of ℒ, and (C) ${\mathscr{K}}$ and ℒ do not overlap,

is my official proposal. So characterised, foundation is both transitive and irreflexive, and is therefore asymmetric. That it is transitive is established in the following proof:

Suppose that ${\mathscr{K}}$ founds ℒ and that ℒ founds ℳ. (i) By clause (A) in (Foundation), it follows that every member of ℳ is grounded in some members of ℒ and every member of ℒ is grounded in some members of${\mathscr{K}}$. Since grounding obeys the cut principle, it follows that every member of ℳ is grounded in some members of ${\mathscr{K}}$. (ii) Suppose for reductio that some member of ${\mathscr{K}}$ is partially grounded in some member of ℳ. Since ℒ founds ℳ, by clause (A) in (Foundation), every member of ℳ is partially grounded in some member of ℒ. Since partial grounding is transitive, it follows that some member of ${\mathscr{K}}$ is partially grounded in some member of ℒ. But given the assumption that ${\mathscr{K}}$ founds ℒ and clause (B) in (Foundation), this is impossible. Hence, no member of ${\mathscr{K}}$ is partially grounded in some member of ℳ. (iii) Suppose for reductio that ${\mathscr{K}}$ overlaps ℳ. Let then F be in both ${\mathscr{K}}$ and ℳ. Since ℒ founds ℳ, by clause (A) in (Foundation) and the fact that F is in ℳ, F is partially grounded in some member of ℒ. But given the assumption that ${\mathscr{K}}$ founds ℒ, clause (B) in (Foundation) and the fact that F is in ${\mathscr{K}}$, this is impossible. Hence, ${\mathscr{K}}$ does not overlap ℳ.

Irreflexivity clearly follows from (C), but also from (A) and (B) taken jointly. The presence of both (B) and (C) does not create redundancy, though, because having just (A) & (B) or just (A) & (C) would yield problems. Keeping just (A) & (C) means opting for (Foundation-4), and we saw that (Foundation-4) does not secure the asymmetry of foundation. Keeping just (A) & (B) is problematic for another reason. Given that a case of foundation is a case where one kind of facts generates another kind of facts, it sounds intuitively correct to say that if a kind ${\mathscr{K}}$ founds a kind ℒ, then ${\mathscr{K}}$ and ℒ do not overlap. Dropping condition (C) in (Foundation) yields a characterisation that fails to guarantee that this is the case. For let ${\mathscr{K}}_{0}$ be the kind *ungrounded fact*, ${\mathscr{K}}_{1}$ the kind *fact grounded in facts in* ${\mathscr{K}}_{0}$, *and not partially grounded in facts outside of* ${\mathscr{K}}_{0}$, and ${\mathscr{K}}_{2}$ the kind *fact grounded in facts in* ${\mathscr{K}}_{1}$. Consider then the kind ${\mathscr{K}}$ that is the union of ${\mathscr{K}}_{0}$ and ${\mathscr{K}}_{1}$ and the kind ℒ that is the union of ${\mathscr{K}}_{1}$ and ${\mathscr{K}}_{2}$ (I suppose for the sake of the argument that these are natural kinds). The characterisation under consideration predicts that ${\mathscr{K}}$ founds ℒ, even though ${\mathscr{K}}$ and ℒ overlap.

### <–fundamentality

Let me now turn to the issue of characterising <–fundamentality. Remember the characterisation put forward at the beginning of Sect. 5:

(<–Fundamentality-1) F *is more fundamental than* G ≡ for some ${\mathscr{K}}$ and ℒ such that F ∈ ${\mathscr{K}}$, F ∉ ℒ, G ∈ ℒ and G ∉ ${\mathscr{K}}$, ${\mathscr{K}}$ founds ℒ.

Given condition (C) in (Foundation), it can be simplified a bit:

(<–Fundamentality-2) F *is more fundamental than* G ≡ for some ${\mathscr{K}}$ and ℒ such that F ∈ ${\mathscr{K}}$ and G ∈ ℒ, ${\mathscr{K}}$ founds ℒ.

This characterisation of <–fundamentality is very natural and simple, but it must be rejected because it does not guarantee that the relation has the right formal properties (remember that I impose as a constraint on any account of <–fundamentality that it should guarantee that the relation is both transitive an irreflexive, and hence asymmetric—see the end of Sect. 2). With (Foundation) in place, (<–Fundamentality-2) is subject to my objection against clause (5) in Bennett’s (MFT), i.e. objection (III). A slightly simplified version of the objection can actually be raised.^18^ Replace the kind *conjunction of atomic facts* by the kind *atomic fact* and the kind *disjunction of conjunctions of atomic facts* by the kind *conjunction of atomic facts*. Then consider the plausible view that the kind *atomic fact* founds the kind *conjunction of atomic facts* and the other plausible view that the kind *biological fact* founds the kind *mental fact*. Then (<–Fundamentality-2) predicts that every conjunction of atomic biological facts is both more and less fundamental than every atomic mental fact.

The previous objection is to the effect that the asymmetry of <–fundamentality is not guaranteed by the proposed characterisation. Here is a further objection, this one to the effect that the proposed characterisation does not secure transitivity. Let ${\mathscr{K}}$ be the kind *human biological fact*, ℒ the kind *human mental fact*, ℒ* the kind *mental fact*, and ℳ the kind *social fact*. The view that ${\mathscr{K}}$ founds ℒ and ℒ* founds ℳ is plausible. Assume the view to be correct and let F be in ${\mathscr{K}}$, G be in ℒ and hence in ℒ*, and H be in ℳ. Assuming (Foundation), (<–Fundamentality-2) predicts that F is more fundamental than G and G is more fundamental than H. Do we have that F is more fundamental than H? Granted that H is a social fact that is not grounded in *human* mental facts but is rather a non-human social fact grounded in mental facts of a very different sort, the reply is plausibly negative.

In the light of these considerations, a radical move suggests itself: assume that distinct kinds of facts do not overlap. Given this assumption, the proposed objections against (<–Fundamentality-2) are ineffective since they crucially involve overlapping kinds. It can actually be *proved* that given the assumption, (<–Fundamentality-2) characterises a relation that is both transitive and irreflexive, and hence asymmetric. That the relation is irreflexive is easy to see. Establishing transitivity requires only a little bit of work:

Suppose F is more fundamental than G and G is more fundamental than H. By (<–Fundamentality-2), there are kinds of facts ${\mathscr{K}}$, ℒ, ℒ* and ℳ such that F ∈ ${\mathscr{K}}$, G ∈ ℒ, G ∈ ℒ*, H ∈ ℳ, ${\mathscr{K}}$ founds ℒ and ℒ* founds ℳ. Given that distinct facts do not overlap, we then have ℒ = ℒ*. Since, as we saw, foundation is transitive, it follows that ${\mathscr{K}}$ founds ℳ. Since F ∈ ${\mathscr{K}}$ and H ∈ ℳ, we can conclude via (<–Fundamentality-2) that F is more fundamental than H.

However, the no-overlap assumption is problematic. Even if we focus, as I argued we should, on *natural* kinds of facts, it is clear that the no-overlap assumption is false: we should recognise that there are, say, both the kind *physical fact* and the kind *microphysical fact*. It might be replied that we should relativise the relation of <–fundamentality to *categorisations*, where a categorisation is defined as a set of non-overlapping kinds. On that account, <–fundamentality would in effect be a 3-place relation rather than a 2-place relation. However, the suggested relativised relation is artificial, because the grouping of kinds into categorisations, that is, into sets of non-overlapping kinds, has no metaphysical significance (in the present context at least). Be that as it may, the initial task was to find an acceptable characterisation of a *2-place* relation of *being more fundamental than* between facts.

At this stage, it is tempting to try to tinker with the right-hand-side of (<–Fundamentality-2), perhaps by invoking universal quantifiers instead of existential quantifiers over kinds, perhaps a mix of both, or by invoking some conditions on kinds, or something that involves both sorts of moves. However, I wish to stay as close as possible to the original Bennettian characterisation, and I accordingly opt for a strategy of “building on top of” (<–Fundamentality-2). Let me use ‘F is quasi-prior to G’ for the condition expressed by the right-hand-side of (<–Fundamentality-2). I argued in effect that one cannot identify <–fundamentality with quasi-priority by giving counterexamples to both the asymmetry and the transitivity of quasi-priority. My proposal is to identify <–fundamentality not with quasi-priority, but with the asymmetric closure of its transitive closure.

Let me spell this out. Where n is a positive integer, say that there is an *n-chain* from F to G iff there are facts F_0_, …, F_n_ such that F_0_ = F, F_n_ = G and for all integers k such that 0 ≤ k ≤ n–1, F_k_ is quasi-prior to F_k+1_. Then say that F is *chained* to G iff there is an n-chain from F to G for some positive integer n. Chaining is the transitive closure of quasi-priority. The proposal is to define <–fundamentality as the asymmetric closure of chaining, that is, to define it as follows:

(<–Fundamentality) F *is more fundamental than* G ≡ F is chained to G and G is not chained to F.

The transitive closure of any relation is transitive. The asymmetric closure of any relation is asymmetric. The asymmetric closure of any transitive relation is itself transitive. Given these three facts, <–fundamentality as characterised above is both asymmetric and transitive (and of course, being asymmetric it is also irreflexive). As a consequence, (<–Fundamentality) is immune from the objections I raised against (<–Fundamentality-2). Note that on the assumption that the kinds of facts are disjoint (an assumption I argued against above), (<–Fundamentality) is equivalent to (<–Fundamentality-2): on that assumption, as we saw, quasi-priority is both transitive and irreflexive; being transitive, it is identical to its transitive closure, namely chaining; and being transitive and irreflexive, it is asymmetric, and as a result it is identical to its asymmetric closure.

The proposed account of <–fundamentality has been designed to fit well with the kind-theoretic conception of fundamentality as described in Sect. 4 and I take it that it does justice to a number of judgments of <–fundamentality that many are inclined to make under that conception. But it arguably does not do justice to *all* such judgments. Consider for instance the view that the biological facts are more fundamental than the mental facts. I guess that many would take it to be correct granted a kind-theoretic conception of fundamentality. However, material that I used against (<–Fundamentality-2) can be used to argue that the view is false given (<–Fundamentality): if F is a conjunction of atomic biological facts and G an atomic mental fact, and if the kind *atomic fact* founds the kind *conjunction of atomic facts* and the kind *biological fact* founds the kind *mental fact*, then by (<–Fundamentality) F is *not* more fundamental than G. Is this a problem?

I do not think so. When we look at the particular example, we see that two forces pull in opposite directions, as it were: the logical features of the facts pull in the direction of G being more fundamental than F, and the “subject matter” of the facts pulls in the direction of F being more fundamental than G. By (<–Fundamentality), these two forces cancel each other, and we get that neither fact is more fundamental than the other. This intuitively makes sense independently from the proposed account: why should the subject matter of the facts prevail over their logical features, or vice versa? Agreed, (<–Fundamentality) may not do justice to the view that the biological facts are more fundamental than the mental facts; but we have a natural account of why the view is incorrect.

Also, it is not clear that what I described as the inclination to endorse the view that the biological facts are more fundamental than the mental facts *is* the inclination to endorse *that very view*. There are several other views in the vicinity which, given plausible ground-theoretic assumptions about physical facts and biological facts, mesh perfectly well with the proposed account of <–fundamentality, for instance:

- The kind *biological fact* founds the kind *mental fact*;
- Every logically simple determinate physical fact is more fundamental than every logically simple determinate biological fact;
- Every logically simple determinate physical fact is more fundamental than every biological fact whatsoever;
- Every biological fact is less fundamental than some physical fact, whereas no biological fact is more fundamental than some physical fact.

It may well be that, in some cases at least, the inclination in question is that of endorsing some such view.

## Characterising = –fundamentality and absolute fundamentality

Given the proposed characterisation of <–fundamentality, how are = –fundamentality and absolute fundamentality to be characterised? For = –fundamentality, I will not be very original. The Litland-Bennett proposal, labelled ‘(AFA)’ in Sect. 3, strikes me as adequate:

(=–Fundamentality) F *is as fundamental as* G ≡ for all facts F*, (i) F* is more fundamental than F iff F* is more fundamental than G, and (ii) F is more fundamental than F* iff G is more fundamental than F*.

(Note that the proposal guarantees that = –fundamentality is an equivalence relation, and hence satisfies the constraint on accounts of that relation that I formulated at the end of Sect. 2.) The case of absolute fundamentality deserves more discussion. The natural proposal, I take it, is to deem a fact absolutely fundamental when it is minimal for the relation of *being more fundamental than*:

(Fundamentality) F *is fundamental* ≡ no fact is more fundamental than F.

Bennett, remember, has another characterisation:

(F) F *is fundamental* ≡ F is ungrounded.

It turns out that given (MFT), these two characterisations are provably equivalent. For suppose F is ungrounded. A quick inspection of (MFT) reveals that on (MFT), if a fact is less fundamental than another fact, then it is grounded. Consequently, given (MFT), F is minimal for <–fundamentality. Conversely, suppose that F is grounded. Then F is partially grounded, and by clause (2) of (MFT) it follows that some fact is more fundamental than F, i.e., that F is not minimal for <–fundamentality.

By contrast, on my characterisation of <–fundamentality, (Fundamentality) and (F) are not provably equivalent. Given (<–fundamentality), being ungrounded provably implies being minimal for <–fundamentality: if a fact F is not minimal for <–fundamentality, then some fact is quasi-prior to it, and this requires that F belong to a kind whose members are all grounded. But being minimal for <–fundamentality does not provably imply being ungrounded. Finding convincing examples to illustrate it is not easy. Here is a tentative suggestion: the fact that God exists. As accompanying assumptions that would guarantee that this fact fits the bill, I suggest the following two: (i) the fact that God exists is grounded in the fact that it is part of His nature that He exists, and (ii) the fact that God exists only belongs to kinds of facts that comprise at least some ungrounded members and hence that are not founded in any other kinds of facts. Assumption (i) is reminiscent of (some versions) of the ontological argument for the existence of God.^19^ In order to secure (ii), more assumptions need to be made. The fact that God exists belongs to the kind *existence fact*. We should accordingly assume that for some object x, the fact that x exists is ungrounded. We might for instance assume that the empty set is such an object. The fact that God exists belongs to the kind *divine fact*. We should accordingly assume that some such fact is ungrounded. We might for instance assume that the fact that it is part of God’s nature that He exists (the fact mentioned as a ground in assumption (i) above) is a fact of that sort. The hope is that whatever kind is invoked, we will always be able to secure, perhaps via extra assumptions, that the kind in question comprises ungrounded facts.

Some might argue that it is a bad feature of my characterisation of absolute fundamentality that it leaves room for there being fundamental yet grounded facts. But I disagree. I have nothing against the view that on *some* conceptions of fundamentality, absolute fundamentality should be equated with ungroundedness. Yet throughout this paper I have been concerned with one particular conception of fundamentality, the kind-theoretic conception; and once this conception is taken for granted, I do not see any problem with the idea that some fundamental facts may be grounded.^20^

## Footnotes

1 In Correia (2021), I explore an opposite view, according to which it is grounding that is characterised in terms of relative fundamentality. This view will not be discussed here. I should mention that at the time of writing that paper I was a bit sceptical about the possibility of formulating an account of relative fundamentality of the sort I put forward in the present paper (see pp. 1280–1281).

2 See for instance Audi (2012: p. 710), Bennett (2011: p. 1), Bennett (2017: p. 106), Dixon (2016: p. 442), Leuenberger (2020: p. 2650), Rosen (2010: p. 112), Schaffer (2009: p. 373), Shumener (2017: p. 2) and Wallner (2021: p. 1260).

3 Werner only implicitly identifies *being fundamental* with *being ungrounded*.

4 I say ‘part of the reason’ partly because I am not opposed in principle to the view that these two ideas may be combined into an account of a unified notion (although I have no idea how this could be done). What I think creates dis-unity is, among other things, the specific way in which these two ideas are put together. See Sect. 3 for the details of Bennett’s characterisation and more on the issue of unity.

5 The idea that reality is hierarchically structured into various layers (or levels) and that entities are more or less fundamental than, or as fundamental as, others depending on which layer they belong to, is admittedly fairly widespread and predates the current massive interest in the notion of grounding (see Kim (2002) for a nice discussion of this idea from the pre-grounding era). The idea that some structures of this sort are generated by grounding (rather than, say, emergence, supervenience or mereological relations) is nowadays also contemplated by a number of philosophers (apart from Bennett (2017), see, *inter alia*, deRosset (2013) and Rabin (2018)).

6 The principle is formulated in this general form for instance by Litland (2013: p. 20). Transitivity proper is a property of one–one relations, satisfaction of the cut principle is the natural correlate for many-one relations.

7 Here and everywhere else below, I use the equivalence symbol ‘≡’ indistinctively to point to stipulative definitions and to point to definitions / characterisations that are intended to capture previously given notions. Context should always determine which meaning the symbol has.

8 See Correia (2010) on the predicate vs operator regimentation.

9 See Fine (2012) on the factive vs non-factive distinction.

10 See Dasgupta (2014) and Litland (2016).

11 Schaffer (2012) proposes counterexamples to the transitivity of partial grounding, but Litland (2013) gives very convincing replies.

12 See p. 161 for the first notion, p. 173 for the second one and ch. 5 for the third one. In clause (1) of (MFT), ‘chain’ means chain of facts related by grounding. The idea that every fact has a unique chain that terminates in ungrounded facts is problematic in more than one way. See Werner forthcoming and Correia forthcoming for discussions.

13 Of course, clauses (2) and (3) are equivalent if we assume, as I did right from the start, that partial grounding is transitive.

14 See for instance Fine (2012: p. 58).

15 In response to an objection against (MFT) put forward by Shumener (2019: pp. 5–6), Bennett (2019: p. 332) suggests adding a subclause to (5) which says that the members of ℒ are same-ranked, where two facts are *same-ranked* iff ‘they are the same number of [grounding] steps from the terminus of their [grounding] chains’. (To be accurate, Shumener and Bennett formulate their points in terms of building rather than grounding.) I ignored this modification, because it annihilates the possibility that clause (5) secures, say, the view that biological facts are more fundamental than mental facts. For on the assumption that every mental fact has a grounding chain with a terminus, the conjunction of two mental facts, which is itself a mental fact, surely has a rank that is higher than the rank of any of these two facts.

16 Bennett (2017: p. 149) gives a similar example to illustrate the fact that an entity may be more fundamental than another one even if the latter is not “built” from the former.

17 The assumption may be false of our world, but it cannot be discarded on a priori grounds (see Rosen, 2010: p. 116).

18 This simplified version of the objection does not seriously threaten (MFT)’s clause (5), because it is implausible to hold that the kind *atomic fact* does not contain both grounded and ungrounded members.

19 But see Glazier (2017) against this kind of grounding claim.

20 My account of absolute fundamentality is not the only account of the notion in ground-theoretic terms that leaves room for the possibility of fundamental yet grounded facts. See Giannotti forthcoming and Correia forthcoming for illustrations. Raven’s (2016) notion of fundamentality as ineliminability is another illustration.
